# T Cells and Gene Regulation: The Switching On and Turning Up of Genes after T Cell Receptor Stimulation in CD8 T Cells

**DOI:** 10.3389/fimmu.2016.00076

**Published:** 2016-02-29

**Authors:** James M. Conley, Michael P. Gallagher, Leslie J. Berg

**Affiliations:** ^1^Department of Pathology, University of Massachusetts Medical School, Worcester, MA, USA

**Keywords:** inducible T cell kinase, TCR signal strength, rheostat, signal transduction, CD8 effector, IRF4

## Abstract

Signaling downstream of the T cell receptor (TCR) is directly regulated by the dose and affinity of peptide antigen. The strength of TCR signaling drives a multitude of T cell functions from development to differentiation. CD8 T cells differentiate into a diverse pool of effector and memory cells after activation, a process that is critical for pathogen clearance and is highly regulated by TCR signal strength. T cells rapidly alter their gene expression upon activation. Multiple signaling pathways downstream of the TCR activate transcription factors, which are critical for this process. The dynamics between proximal TCR signaling, transcription factor activation and CD8 T cell function are discussed here. We propose that inducible T cell kinase (ITK) acts as a rheostat for gene expression. This unique regulation of TCR signaling by ITK provides a possible signaling mechanism for the promotion of a diverse T cell repertoire in response to pathogen.

## Introduction

T cells bind antigen through their T cell receptor (TCR), activating a signaling cascade that leads to proliferation, differentiation, and pathogen clearance. The affinity of a TCR for its cognate antigen has been directly linked to the regulation of thymic selection and the T cell immune response. Upon receptor activation, tyrosine kinases are phosphorylated, creating signaling complexes at the plasma membrane that activate downstream signal transduction pathways. In response to activation, the T cell reorganizes its cytoskeleton, changes its metabolism, and alters its gene expression. This review focuses on the signaling pathways that contribute to changes in gene expression. The three main pathways activated through the TCR that control transcription are the MAPK, NF-κB, and calcium pathways. These pathways dramatically alter the expression and nuclear localization of various transcription factors that directly regulate genes involved in T cell activation (1, 2).

T cells recognize peptide antigens displayed by the major histocompatibility complex (pMHC) on antigen-presenting cells (APCs). The interactions between the TCR and pMHC have been extensively characterized in the context of thymic selection and in the immune response to infection (3). In response to viral infection, CD8 T cells recognize antigen from APCs in the lymphoid organs, undergo clonal expansion, migrate to the site of infection, kill infected cells, and then die by apoptosis. Some of the CD8 T cells will survive this population contraction to form a memory pool (4).

Because T cells are polyclonal and contain variable TCRs, they are able to recognize a wide variety of peptide ligands. These ligands can have variable binding affinities for the TCR, creating a range of ligand-binding kinetics (5). This variability contributes to the process of graded TCR signaling, where altered peptide ligands (APLs) can generate different signaling outcomes depending on the binding kinetics of the TCR and pMHC. This review highlights recent advances in the field, which connect proximal TCR signaling and transcription factor activation to changes in CD8 T cell function.

## Graded TCR Stimulation and Signal Transduction

The effect of APLs on the proximal signaling events downstream of the TCR has been extensively examined. In the basal state, the ITAMs of the TCR zeta chains are phosphorylated and bound to inactive Zap70. The dwell time of an agonist–peptide interaction with the TCR is significantly longer than self-peptide interactions, creating a higher probability that a CD8 coreceptor bound to active Lck can be recruited and bind pMHC. This interaction increases complex stability and creates an opportunity for Lck to activate Zap70 by first phosphorylating Y319, relieving the autoinhibited conformation. Further activation of other tyrosine residues increases Lck and Zap70 interactions and further initiates both kinases to create the downstream LAT signaling complex (6). These proximal signaling events are absolutely required for downstream TCR signal transduction (7, 8). Using an engineered mutant allele of Zap70, which is sensitive to a small molecule kinase inhibitor, a sharp threshold of signaling was observed downstream of the kinase. Using Nur77–GFP as a readout for TCR signaling, Zap70 was determined to be required for signaling and cell division. This requirement was independent of both the strength of the pMHC–TCR interaction and IL-2 (9).

Not all pathways downstream of the TCR are similarly affected by antigen avidity. The calcium signaling pathway has been shown to be disproportionately affected by weak antigen stimulation (10–12). The MAPK and NF-κB pathways, while activated by the TCR, have a threshold level of activation that is less affected by weak stimuli. The MAPK pathway starts with Ras activation and subsequently leads to downstream Erk activation and the formation of the transcription factor dimer AP-1. In T cells, Ras is activated downstream of the TCR by RasGRP or SOS. Initially, RasGRP is activated by binding diacylglycerol (DAG), a byproduct of PtdIns(4,5)P_2_ (PIP_2_) cleavage, followed by phosphorylation by protein kinase c (PKC) at the plasma membrane. This process is dependent on TCR signal transduction and ultimately on activation of phospholipase C (PLCγ). PLCγ activation gradually increases the local concentration of RasGRP at the LAT signaling complex. While this initial signaling event is graded in nature, it rapidly triggers a positive feedback loop. SOS acts as the integration point in this feedback loop; through allosteric binding, SOS substantially increases the GTPase activity of RasGTP (13). This feedback loop ensures that low-affinity TCR ligation can trigger robust Ras activation. Indeed, a digital signal response is observed with p-Erk, with different affinity antigens triggering maximal pathway activation. CD69 was also observed to have this digital response and its expression was highly dependent on the MAPK pathway (13, 14).

A similar signaling effect was observed in the NF-κB pathway. Jurkat cells stimulated with different concentrations of anti-CD3 antibody were able to activate T cells in a graded manner. However, looking at IκBα degradation, the pathway was digital in nature with cells forming a bimodal population (15). Using a mutant form of the OT-I TCR (bTMD) having intact antigen-binding capacity but dampened signaling, a defect in PKC recruitment to the immunosynapse was observed shortly after stimulation. This defect leads to impaired CD8 memory formation. However, only a signaling defect in the NF-κB pathway was observed downstream of the TCR suggesting that a small signaling defect in this pathway could impair CD8 differentiation (16). The diverse functions of NF-κB signaling downstream of the TCR have been extensively reviewed (17).

The quality of the calcium signaling response is highly sensitive to TCR antigen affinity. Specifically, the influx oscillation patterns of this cation are highly mutable (10, 11, 18, 19). Using calcium indicator dyes to measure changes in intracellular ion concentration, changes in both the amplitude and duration of calcium influx have been observed upon stimulation with antigens of different affinities (10, 19). Low-affinity pMHC does not trigger robust calcium influx in multiple *in vitro* T cell experiments. This has been linked to the regulation of calcium egress from the endoplasmic reticulum, a process that is mediated by the inositol trisphosphate receptor (IP3R) (10). In CD4 T cells, the time of onset for calcium flux was directly proportional to the affinity of the antigen. Weaker pMHC stimulation also reduced the magnitude of the influx peak and the duration of the influx (11). Calcium oscillation patterns have been directly linked to nuclear signaling in T cells with a non-linear relationship between [Ca^2+^] and transcription factor activation observed (20). Specifically, high frequency oscillation drove both NFAT and NF-κB activation, whereas low frequency oscillation drove only NF-κB activation. This, combined with differences in nuclear dwell time (>15 m for NF-κB and <10 m for NFAT), accounts for variable pathway activation in response to changes in calcium influx (21).

The calcium-sensitive transcription factor NFAT is well known for its contribution to T cell activation, specifically through the production of IL-2 and prosurvival factors. NFAT-binding partners are also critical for T cell activation. Recently, the genetic targets of NFAT were further characterized by comparing constructs that allowed or ablated binding with its most common partner, AP-1. A subset of genes critical for the promotion of CD8 T cell exhaustion were highly dependent on NFAT, with transcription occurring in the absence of AP-1 (22). The amount of time that NFAT spends inside the nucleus is critical for specific transcriptional targets. Using multiphoton intravital microscopy, the half-lives of NFAT nuclear import and export were calculated to be ~1 min for import and ~20 min for export. The tolerance gene *Egr2* was sensitive to short low-affinity antigen stimulation, while the effector gene *Ifng* needed prolonged high-affinity stimulation (23).

**Figure 1:**
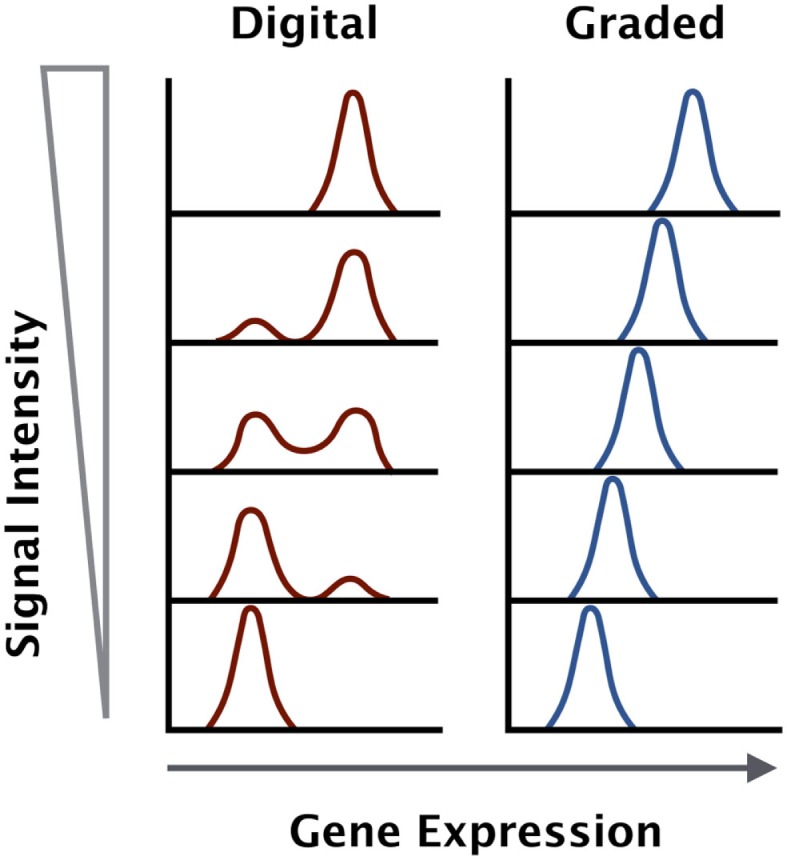
**Gene expression outputs downstream of the TCR can be digital or graded in nature**. Some genes are upregulated to a maximal level and the increase in antigen dose will increase the percentage of positive cells within a given population (digital), while other genes increase in expression in direct proportion to the antigen dose and affinity across the entire population (graded).

The signaling data suggest that certain factors like CD69 and IL-2 are digital in nature, while others like interferon regulatory factor 4 (IRF4) are analog in nature. The strength of TCR stimulus, whether through increased dose or affinity of antigen, is not proportional to the output of the MAPK and NF-κB signaling pathways. However, the activation marker IRF4 is proportional to the strength of stimulus (Figure 1).

## Graded TCR Signaling and Transcription Factors

In addition to the canonical transcription factors AP-1, NF-κB, and NFAT, which are activated by the TCR, CD8 T cells rely on a diverse set of transcription factors that drive different functions. Recently, the balance of Blimp-1, T-Bet, Bcl-6, and eomesodermin has been linked to changes in CD8 differentiation with high TCR signal strength driving T-Bet and Blimp-1 expression, leading to terminal effector differentiation (24–26). Changes in transcription have also been linked to TCR affinity, specifically the transcription factor IRF4 (27). While it shares homology with other family members like IRF3, Type 1 IFN does not regulate IRF4 (28). Rather, its expression is directly regulated by antigen receptors in both T cells and B cells. Undetectable in naive lymphocytes, IRF4 is rapidly upregulated by antigen stimulation and the magnitude of expression is proportional to the strength of stimulus (29). The NF-κB family transcription factor Rel directly regulates *Irf4* at the promoter locus (30). In CD8 T cells, IRF4 expression directly impacts clonal expansion and viral clearance. Specifically, the amount of IRF4 expressed in the CD8 T cell contributed to the expansion of short-lived effector cells (SLECs) (31–33). These terminally differentiated T cells are critical for pathogen control and require robust proliferation before the whole population is eliminated through apoptosis. IRF4 contributes to SLEC functionality by regulating multiple genes associated with glycolysis (32, 34). CD8 T cells rapidly shift their metabolism in response to antigen and similar cancer cells, they use glycolytic pathways to increase glucose import providing energy and macromolecules for biomass expansion and division (35, 36). IRF4 can also act as a repressor and binds sites in genes associated with apoptosis and cell cycle inhibition, supporting its important role in T cell survival and expansion (33). Directly comparing *Irf4*^+/−^ with *Irf4*^−/−^ and WT mice, viral clearance and SLEC expansion were directly proportional to the maximum level of IRF4 expression. The haplosufficient mice had approximately twofold to fivefold less IRF4 production, a reduction that was mirrored in their ability to clear infection (31).

The frequent binding partner of IRF4, BATF, has also been linked to CD8 T cell expansion. However, unlike IRF4, the levels of expression are not as critical. The two transcription factors are regulators of T-bet and Blimp1 expression, the two transcription factors that drive effector differentiation (37). Interestingly, BATF was determined to act as a “persistence detector” of antigen signaling. Maximum BATF-mediated effector differentiation was only triggered by durable antigen signaling. Late in the T cell response, BATF could also repress critical genes like perforin and granzyme B, dampening the late effector response (37).

Another factor associated with TCR affinity is Nur77, a transcription factor primarily associated with apoptosis (38). Nur77 is often used as a reporter for TCR activation with its expression proportional to antigen avidity. The amount of Nur77 expression correlates with thymic selection, with negatively selected thymocytes having higher levels than their positively selected counterparts (39, 40). Nur77 is rapidly upregulated after antigen stimulation, and its expression is dependent solely on TCR signaling. Using a Nur77–GFP reporter mouse, the levels of GFP were proportional to the affinity of TCR ligand in the OT-I model system, showing a graded expression similar to IRF4 (39).

## ITK and Graded TCR Signal Strength

Downstream of Lck, the LAT signaling complex is critical for downstream signaling pathway activation. Inducible T cell kinase (ITK) is a Tec family tyrosine kinase, which is activated by Lck and phosphorylates PLCγ. Unlike its sister kinase Bruton’s tyrosine kinase (BTK), ITK’s expression is largely limited to T cells, and its catalytic activity is in the weak micromolar range. In resting T cells, ITK resides in the cytosol. Upon activation, the kinase is recruited to the plasma membrane through its pleckstrin homology (PH) domain, which binds PtdIns(3,4,5)P_3_ (PIP_3_). ITK also associates with the LAT–SLP76 complex through its SH2 and SH3 domains, creating a signaling complex that is directly dependent on upstream Lck and Zap70 signaling. ITK is directly phosphorylated by Lck and subsequently undergoes *cis*-autophosphorylation. Then, ITK is able to phosphorylate the lipase PLCγ that cleaves PIP_2_ in the plasma membrane, generating the secondary messengers IP3 and DAG. These secondary messengers primarily activate the downstream MAPK, NF-κB, and calcium signaling pathways (41–43).

**Figure 2:**
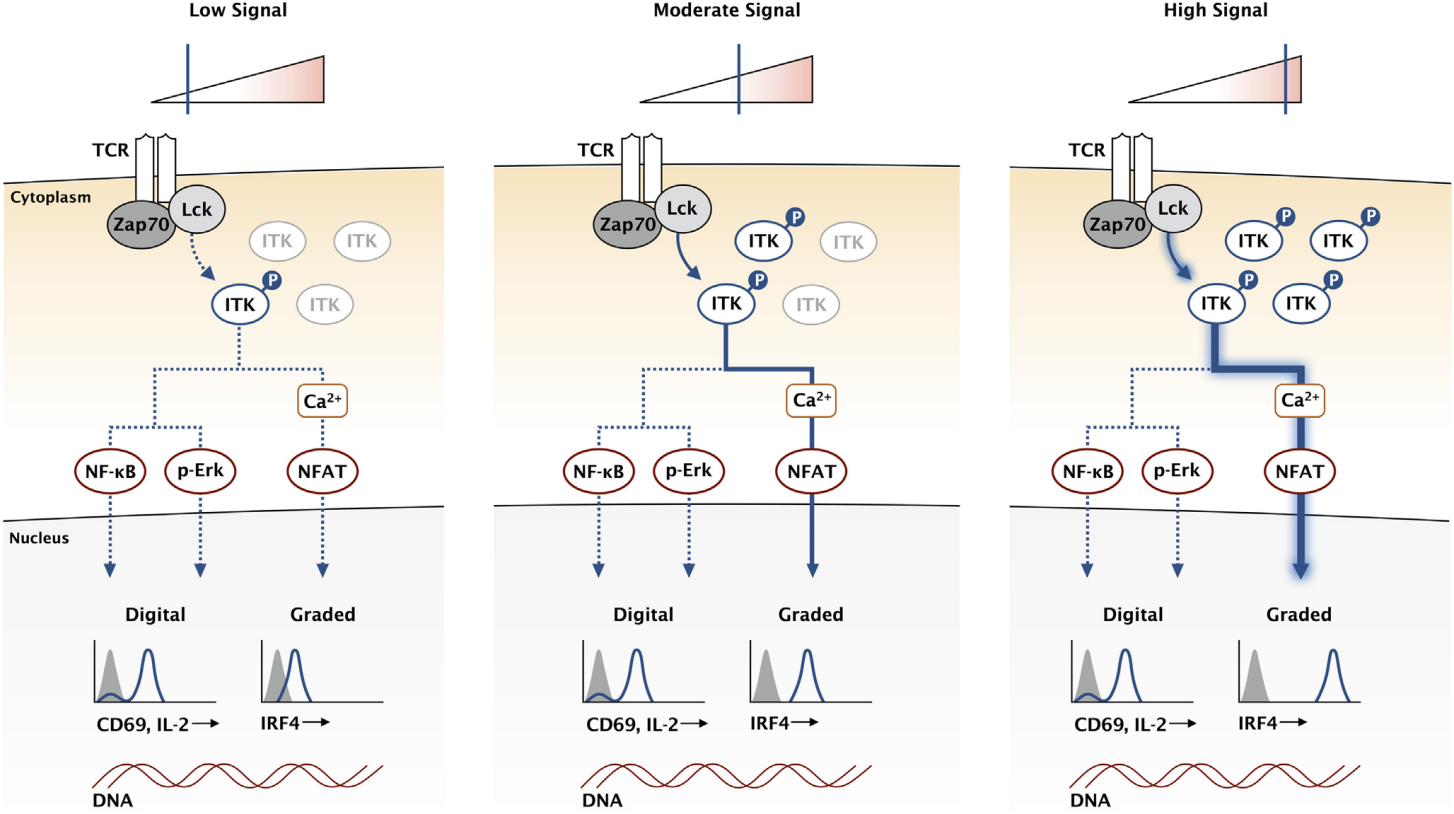
**ITK acts as a rheostat for the TCR signaling pathway**. While the expression of genes like CD69 and IL-2 is not directly proportional to the signaling input at the TCR, other genes like IRF4 have a graded response where expression continues to increase with higher affinity TCR stimulation. High-affinity antigens increase the duration of TCR binding, increasing the stability of activated Lck and Zap70, which amplifies downstream signaling by creating a larger pool of activated LAT signaling complexes. This creates a larger pool of activated ITK, represented here for simplification without the other signaling components in the pathway. We propose that the calcium signaling pathway is uniquely sensitive to ITK and can provide a mechanism for graded IRF4 expression.

Unlike the upstream Src kinases, ITK is not required for T cell activation. Indeed, *Itk-*deficient T cells are capable of proliferation and cytokine production in response to antigen stimulation (44, 45). Mice lacking ITK are also able to clear pathogens in multiple infection models (46–48). However, *Itk*-deficient T cells are not normal, and a diminished response is observed rather than an ablated response. While the MAPK pathway is only modestly impaired in *Itk*-deficient T cells, the magnitude of calcium flux is greatly reduced in response to antigen (12). This reduction in signaling correlates with a reduction in CD8 clonal expansion in response to infection (49). A key set of experiments directly compared kinase dead mutants of Lck and ITK in activated T cells (50). While both mutants inhibited calcium flux in the T cells, the kinases had very different effects on the whole population. The Lck mutants ablated calcium signaling in a proportion of the T cells, while ITK mutants dampened the calcium response of each cell in the entire population. This suggests a digital regulation of calcium signaling through Lck and an analog regulation through ITK. We propose that ITK and the calcium signaling pathway are critical for maintaining the analog nature of this TCR output (Figure 2).

## Graded TCR Signaling and CD8 T Cell Function

CD8 T cell development and function have been studied using the OT-I transgenic mouse model. The OT-I TCR recognizes the SIINFEKL peptide (OVA), an antigen derived from chicken ovalbumin (51). These OT-I T cells are MHC class I restricted, creating a large population of naive CD8 T cells that recognize the Ova antigen. The OT-I model provides an excellent system to study TCR signal strength because of the exquisitely high-affinity OVA peptide ligand. APLs with single amino acid substitutions greatly reduce TCR-binding affinity while maintaining equivalent MHC-binding capacity. Scientists have also engineered a variety of pathogens to express these APLs, providing excellent infection models to study TCR signal strength *in vivo*. In the context of CD8 T cell development in the thymus, graded TCR signal strength drives the balance between positive and negative selection, a process that can directly impact autoimmune disease (52–54).

A series of studies have looked at CD8 T cell activation *in vivo* in response to infection and have teased apart contributions between antigen interactions and environmental inflammatory signals. While T cell expansion was directly proportional to antigen avidity, cytolytic function was more dependent on inflammatory signals (55, 56). Using peptide-pulsed dendritic cells with or without *Listeria monocytogenes* infection, granzyme B and IFN-γ production were only seen in the context of infection. However, IL-2 and the chemokine receptor CCR7 were regulated in the absence of inflammation, and their expression was directly proportional to the avidity of the antigen. The high-affinity IL-2 receptor (CD25) was driven by both signals with its expression proportional to both dose and affinity of the stimulus (55, 57). These *Listeria* experiments show that certain aspects of T cell function are driven by the affinity of the pMHC–TCR interaction, while others are driven by cytokine activation. Low-affinity antigen stimulation downregulated CCR7 earlier than high-affinity stimulation and the T cells were able to migrate out of the periarteriolar lymphocyte sheaths (PALS) at an earlier time, creating a larger proportion of T cells in the blood and the red pulp of the spleen. The high affinity stimulated T cells retained CCR7 expression out to 4 days and did not migrate out of the PALS. Effects on T cell migration and tissue infiltration were also observed in the context of autoimmunity (57). In a model where membrane-bound ovalbumin (mOVA) is under the control of the rat insulin promoter (RIP), OT-I T cells were transferred into mice after immunization with high- and low-affinity peptide antigen. Only T cells stimulated with high-affinity peptide were able to infiltrate the pancreas and migrate to the site of Ova antigen, a process that was dependent on the integrin VLA-4.

The altered expression in both magnitude and kinetics of CD25, CD69, and CCR7 shows that antigen affinity directly affects IL-2 signaling and lymph node egress. This suggests that T cells stimulated with high-affinity antigen are able to sequester more IL-2 from the environment and remain in the lymph node, thereby allowing them a longer time to receive stimulation. This delay in egress contributes to a more robust clonal expansion, a process that is directly proportional to antigen avidity. Signaling through the IL-2 receptor is required for CD8 memory formation (58). Mice lacking IL-2 receptors in mature CD8 T cells, mixed chimeras that retain a normal population of T regulatory cells, were able to mount a normal primary response to LCMV infection. However, the secondary response was impaired. Thus, IL-2 signaling is required during the primary response to produce a functional memory pool (59). IL-2 can also promote proliferation of T cells stimulated by low-affinity antigen. Using an *in vitro* coculture system of P14 and OT-I CD8 T cells, P14 T cells stimulated with high-affinity GP33 peptide could produce IL-2 and trigger increased proliferation in neighboring OT-I T cells that were stimulated with the low-affinity G4 peptide. The IL-2 is required within 20–30 h of antigen stimulation and PI3K signaling through the IL-2 receptor cooperates with TCR signaling to promote cell cycle entry (60). IL-2 is not the only secondary signal that cooperates with TCR activation, a wide variety of costimulatory pathways interplay with graded TCR signaling to promote diverse CD8 T cell functions (61). The regulation of T cell memory has been extensively linked to a combination of graded TCR signaling and costimulation using multiple transgenic and pathogen models (24).

Affinity of the antigen has also been linked to regulation of asymmetric cell division with proximal daughter T cells becoming SLECs (57, 62). High-affinity interactions led to a distinct progeny after the first division with proximal daughters containing the immunosynapse and increased glycolytic capacity. In contrast, low-affinity TCR interactions did not lead to this phenotype (57). Graded TCR signaling controls many aspects of CD8 T cell differentiation. The signaling mechanisms that connect graded TCR stimulation with these changes in differentiation while not completely understood have been extensively examined in the recent years.

## Conclusion and Future Directions

The data generated with the ITK knockout mice suggest that ITK, while not required for pathogen clearance, can act as a rheostat, capable of amplifying or reducing the magnitude of signaling downstream of the TCR. This tuning of signaling can have effects on multiple processes including T cell development and T cell differentiation in response to infection. Indeed, *Itk*-deficient mice have alterations in T cell development with an increase in innate-like T cells exiting the thymus (63). In response to LCMV infection, *Itk*-deficient CD8 T cells had a defect in expansion, very similar to the defect that was seen in *Irf4* haplosufficient mice (31, 49). Also, expression of IRF4 is highly dependent on ITK activity with a dose proportional reduction observed between IRF4 and ITK inhibition. Our hypothesis is that certain factors like IRF4 depend on TCR signaling for expression, which can be fine-tuned by the rheostat ITK. This can lead to changes in CD8 effector function, providing a link between ITK and CD8 T cell fate. One possible mechanism for this unique level of control is through the calcium pathway. Teasing apart contributions of the different signaling pathways on graded transcription factor expression remains to be done. Directly measuring calcium ion flux in single T cells in the context of graded TCR signaling *in vivo* will determine whether calcium signaling alone drives the rheostat activity of ITK. It is also unknown what other genes are regulated in a similar fashion as IRF4. Future experiments will further elucidate the mechanisms connecting graded TCR signaling to changes in transcription and CD8 T cell function.

## Author Contributions

JC was the principal writer of this mini review. MG contributed significantly to the design of the figures. All work was done under the direct supervision of LB.

## Conflict of Interest Statement

The authors declare that the research was conducted in the absence of any commercial or financial relationships that could be construed as a potential conflict of interest.
